# Trehalose treatment suppresses inflammation, oxidative stress, and vasospasm induced by experimental subarachnoid hemorrhage

**DOI:** 10.1186/1479-5876-10-80

**Published:** 2012-04-30

**Authors:** Ryosuke Echigo, Nobuyuki Shimohata, Kensuke Karatsu, Fumiko Yano, Yuko Kayasuga-Kariya, Ayano Fujisawa, Takayo Ohto, Yoshihiro Kita, Motonao Nakamura, Shigeki Suzuki, Manabu Mochizuki, Takao Shimizu, Ung-il Chung, Nobuo Sasaki

**Affiliations:** 1 Laboratory of Veterinary Surgery, Graduate School of Agricultural and Life Sciences, The University of Tokyo, 1-1-1 Yayoi, Bunkyo-ku, Tokyo, 113-8657, Japan; 2 NEXT21 K.K., 3-38-1 Hongo, Bunkyo-ku, Tokyo, 113-0033, Japan; 3 Center for Disease Biology and Integrative Medicine, Faculty of Medicine, The University of Tokyo, 7-3-1 Hongo, Bunkyo-ku, Tokyo, 113-0033, Japan; 4 Department of Bioengineering, Graduate School of Engineering, The University of Tokyo, 2-11-16 Yayoi, Bunkyo-ku, Tokyo, 113-8656, Japan; 5 Department of Biochemistry and Molecular Biology, Faculty of Medicine, The University of Tokyo, 7-3-1 Hongo, Bunkyo-ku, Tokyo, 113-0033, Japan

**Keywords:** Trehalose, Subarachnoid hemorrhage, Cerebral vasospasm, Inflammatory response, Oxidative stress

## Abstract

**Background:**

Subarachnoid hemorrhage (SAH) frequently results in several complications, including cerebral vasospasm, associated with high mortality. Although cerebral vasospasm is a major cause of brain damages after SAH, other factors such as inflammatory responses and oxidative stress also contribute to high mortality after SAH. Trehalose is a non-reducing disaccharide in which two glucose units are linked by α,α-1,1-glycosidic bond, and has been shown to induce tolerance to a variety of stressors in numerous organisms. In the present study, we investigated the effect of trehalose on cerebral vasospasm, inflammatory responses, and oxidative stress induced by blood *in vitro* and *in vivo*.

**Methods:**

Enzyme immunoassay for eicosanoids, pro-inflammatory cytokines, and endothelin-1, and western blotting analysis for cyclooxygenase-2, inducible nitric oxide synthase, and inhibitor of NF-κB were examined in macrophage-like cells treated with hemolysate. After treatment with hemolysate and hydrogen peroxide, the levels of lipid peroxide and amounts of arachidonic acid release were also analyzed. Three hours after the onset of experimental SAH, 18 Japanese White rabbits received an injection of saline, trehalose, or maltose into the cisterna magna. Angiographic and histological analyses of the basilar arteries were performed. In a separate study, the femoral arteries from 60 rats were exposed to fresh autologous blood. At 1, 3, 5, 7, 10, and 20 days after treatment, cryosections prepared from the femoral arteries were histologically analyzed.

**Results:**

When cells were treated with hemolysate, trehalose inhibited the production of several inflammatory mediators and degradation of the inhibitor of NF-κB and also suppressed the lipid peroxidation, the reactive oxygen species-induced arachidonic acid release *in vitro*. In the rabbit model, trehalose produced an inhibitory effect on vasospasm after the onset of experimental SAH, while maltose had only a moderate effect. When the rat femoral arteries exposed to blood were investigated for 20 days, histological analysis revealed that trehalose suppressed vasospasm, inflammatory response, and lipid peroxidation.

**Conclusions:**

These data suggest that trehalose has suppressive effects on several pathological events after SAH, including vasospasm, inflammatory responses, and lipid peroxidation. Trehalose may be a new therapeutic approach for treatment of complications after SAH.

## Background

Aneurysmal subarachnoid hemorrhage (SAH) is a serious condition which often leads to high mortality and morbidity [1]. Ischemic injury due to cerebral vasospasm is a major cause of brain damage after SAH. However, cerebral vasospasm is only considered one of the underlying causes of SAH, and does not represent all of the associated clinical manifestations. A recent study also demonstrated that recovery of vasoconstriction by an endothelin antagonist did not necessarily contribute to improved clinical outcome [2], and additional mechanisms of secondary injury besides vasospasm have been suggested [3].

SAH occurs after the rupture of an aneurysm on the cerebral artery wall. Erythrocyte cytosol is released into the subarachnoid space through hemolysis of erythrocytes. Oxyhemoglobin or a related high molecular weight compound in erythrocyte cytosol can elicit a wide range of stress responses, including activation of inflammatory responses and production of inflammatory cytokines [4]. These actions are partially regulated by the NF-κB signaling pathway. The NF-κB signaling pathway is essential for host defense and inflammatory responses to extracellular stimuli, but also has been associated with production of inflammatory mediators after SAH [5]. It is also known that the production of a class of arachidonic acid-derived eicosanoids is enhanced in patients with SAH [6]. In addition, oxidative stress, including lipid peroxidation, can occur following SAH due to excessive free radicals generated by oxyhemoglobin and enzymatic reactions, and plays important roles in the pathogenesis of acute brain injury, development of vasospasm, and breakdown of the blood brain barrier [7]. In support, overexpression of antioxidant enzyme has been shown to attenuate early brain injury after SAH [8].

A number of human and animal studies have been performed on irrigation of the subarachnoid space for the removal of blood clots, percutaneous transluminal angioplasty, anti-inflammatory agents including nonsteroidal anti-inflammatory drugs and glucocorticoids, and antioxidants including radical scavengers and lipid peroxidation inhibitors [4,9,10]. However, there are still no definitive treatments for complications including vasospasm after SAH. As the underlying causes of the complications following SAH are multifactorial, effective treatment will likely require a combination of approaches including clearance of blood and the use of anti-oxidant reagents and anti-inflammatory reagents, or a reagent with pleiotropic effects.

Trehalose is a non-reducing disaccharide in which two glucose units are linked by an α, α-1, 1-glycosidic bond. Trehalose has multiple functions that distinguish it from other common disaccharides, including a protective action against stressors such as desiccation, reactive oxygen species (ROS), and cold [11,12]. In addition, recent reports showed that trehalose could prevent inflammatory responses induced by endotoxic shock *in vivo* and *in vitro *[13,14]. As such, trehalose is considered a potentially powerful therapeutic agent for various diseases, involving oxidative stress, desiccating conditions, and chronic inflammation. Trehalose is also considered to have a high safety profile as a trehalose-containing organ preservation solution is used in clinical lung transplantation [15]. Furthermore, clinical application of trehalose has been attempted for the cryopreservation of platelets, dry eye syndrome, and oral dryness caused by dental treatment [16-18].

In the present study, we examined whether trehalose could suppress oxidative stress, inflammatory responses, and cerebral vasospasm after SAH.

## Methods

### Materials, animals and cell culture

Trehalose and maltose were purchased from Wako (Osaka, Japan). The percentage of trehalose in solution using the *in vitro* and *in vivo* experiments was selected based on our preliminary data (data not shown).

Lipopolysaccharide (LPS) (from *Escherichia coli* O111:B4) was from Sigma (St. Louis, MO, USA), and anisomycin (from *Streptomyces griseolus*) was from Calbiochem (San Diego, CA, USA).

Male Japanese White rabbits, male Wistar rats, and male Sprague–Dawley (SD) rats were purchased from Japan SLC (Hamamatsu, Japan). The design of the animal study was approved by the Animal Care Committee of the Graduate School of Agricultural and Life Sciences, The University of Tokyo.

We investigated the effect of trehalose on blood-induced inflammatory responses in macrophage cells and human umbilical vein endothelial cells (HUVECs). These cells play important roles in inflammation after SAH [19-22]. The hemolysate (see below) was used for *in vitro* studies on blood-induced inflammation. The murine macrophage cell line RAW 264.7 was obtained from the RIKEN Cell Bank (Ibaraki, Japan). RAW 264.7 cells were cultured in Dulbecco’s Modified Eagle’s Medium (DMEM) (Sigma) supplemented with 10% (v/v) fetal bovine serum (FBS) (Gibco, Carlsbad, CA, USA) and 1% (v/v) penicillin and streptomycin (Sigma). HUVECs were obtained from Lonza (Basel, Switzerland). HUVECs were grown in endothelial basal medium supplemented with growth factors and FBS (EBM-2; Lonza).

### Preparation of hemolysate

Hemolysates from Wistar rats (weighing approximately 250 g) were prepared according to a previously described method [23], with slight modifications. The erythrocyte fraction was washed three times with saline, and then erythrocytes were divided into three parts and suspended in either saline, 10% (w/v) trehalose (final concentration, 7.5%), or 10% (w/v) maltose (final concentration, 7.5%), and then gently sonicated for 30 s. After centrifugation, the supernatants were collected as erythrocyte hemolysates and stored at −80°C until use. Two absorbance peaks at 540 and 576 nm were spectrophotometrically measured in hemolysate samples, confirming the presence of oxyhemoglobin.

### Enzyme immunoassay for eicosanoids, pro-inflammatory cytokines, and endothelin-1

RAW 264.7 cells or HUVECs were incubated with or without 10% hemolysate in the medium mixed with an equal part of saline, 10% trehalose, or 10% maltose for 8 h or 18 h, and then washed three times with fresh medium. After incubation for 1 h, the culture media were collected and centrifuged at 15,000 rpm for 10 min. The supernatants were collected for tests. The supernatant concentrations of prostaglandin E_2_ (PGE_2_), cysteinyl leukotriene, human endothelin-1, tumor necrosis factor-α (TNF-α), interleukin-6 (IL-6), interleukin-1α (IL-1α), and interleukin-1β (IL-1β) were measured using enzyme immunoassay (EIA) kits (the first two with kits from Cayman, Ann Arbor, MI, USA; the remainder from R&D Systems, Minneapolis, MN, USA) according to the manufacturer’s instructions.

### Arachidonic acid release assay

RAW 264.7 cells were pre-incubated for 24 h in basal medium containing 1 μCi [^3^H]-arachidonic acid/ml (PerkinElmer Life Sciences, Boston, MA, USA). The cells were washed three times with Tyrode solution containing 10 mM HEPES-NaOH (pH 7.4) and 0.1% fatty acid-free bovine serum albumin (Sigma) (wash buffer) and incubated at 37°C for 3 h in wash buffer with 10% hemolysates, 1.5 mM H_2_O_2_, or the corresponding vehicle. At the end of the 3 h incubation period, the incubation medium was removed and centrifuged at 10,000 rpm. The supernatants were collected for tests. To determine the radioactivity of released arachidonic acid, aliquots of the supernatants were placed in scintillation vials containing scintillation fluid, and radioactivity was measured using a liquid scintillation counter LS6500 (Beckman Coulter, Fullerton, CA, USA). To determine the radioactivity of incorporated arachidonic acid, the cells that remained attached to the plates were lysed using 2% NP-40 substitute solution, and the cell lysate was utilized for scintillation counting. The quantity of phospholipase A_2_ (PLA_2_) activity was shown as the total radioactivity released per unit of incorporated radioactivity.

### Western blotting analysis for cyclooxygenase-2, inducible nitric oxide synthase, and IκB-α

**Figure 1:**
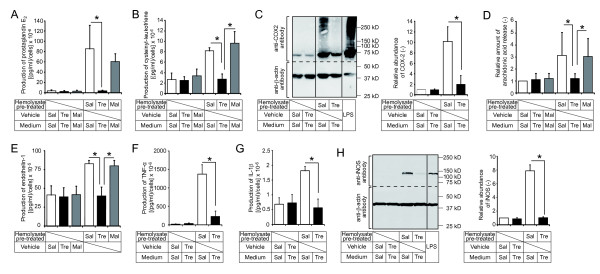
**Effects of trehalose on inflammatory responses in cultured cells.** As indicated in the graphs, culture media, hemolysate, or both contained saline (Sal), 5% trehalose (Tre), or 5% maltose (Mal). (**A, B**) Effect of trehalose on the induction of eicosanoids in hemolysate-treated cells. RAW 264.7 cells were treated with or without 10% hemolysates for 8 h. Prostaglandin E_2_ (PGE_2_) (**A**) and cysteinyl leukotriene (**B**) levels were measured by enzyme immunoassay (EIA). n = 4 (A) and n = 5 (B) in each group, respectively. (**C**) Effect of trehalose on production of cyclooxygenase-2 (COX-2) in hemolysate-treated cells. RAW 264.7 cells were treated with 10% hemolysates, vehicle, or 20 ng/ml LPS (used as a positive control) for 8 h. The amounts of COX-2 and β-actin (used as an internal control) were analyzed by western blotting. The graph shows the band intensities of COX-2, adjusted to the corresponding β-actin bands and calculated as intensity ratio relative to that of the saline control. n = 5 in each group. (**D**) Effect of trehalose on the release of arachidonic acid in hemolysate-treated cells. RAW 264.7 cells were prelabeled with [^3^H]-arachidonic acid and then treated with or without 10% hemolysate for 3 h. The radioactivity was measured to quantify arachidonic acid release. n = 7 in each group. (**E**) Effect of trehalose on the induction of endothelin-1 in hemolysate-treated cells. Human umbilical vein endothelial cells (HUVECs) were treated with or without 10% hemolysates for 18 h. Endothelin-1 levels were measured by EIA. n = 5 in each group. (**F, G**) Effect of trehalose on the induction of pro-inflammatory cytokines in hemolysate-treated cells. RAW 264.7 cells were treated with or without 10% hemolysates for 8 h. Tumor necrosis factor-α (TNF-α) (**F**) and interleukin-1β (IL-1β) (**G**) levels were measured by EIA. n = 3 (F) and n = 4 (G) in each group, respectively. (**H**) Effect of trehalose on the production of inducible nitric oxygen species (iNOS) in hemolysate-treated cells. RAW 264.7 cells were treated with vehicle, 10% hemolysates, or 20 ng/ml LPS for 8 h. Amounts of iNOS and β-actin were analyzed by western blotting. The graph shows the band intensities of iNOS, adjusted to the corresponding β-actin bands and calculated as intensity ratio relative to that of the saline control. n = 7 in each group. Whole experiments were repeated more than three times. All data are shown as means ± SD. *P < 0.05 (Kruskal-Wallis test).

RAW 264.7 cells were incubated with or without 10% hemolysate, 20 ng/ml LPS, or 10 μM anisomycin in medium mixed with an equal part of saline or 10% trehalose for 8 h or at the time points indicated in figure 1 legends. After washing with ice-cold PBS, cells were collected and lysed using TNE buffer (10 mM Tris–HCl pH 7.8, 150 mM NaCl, 1 mM EDTA, and 1% NP-40) containing 100 μg/ml aprotinin, 2 mM Na_3_VO_4_, 10 mM NaF, and protease inhibitor cocktail (Roche, Basel, Switzerland). Immunoblotting with the specific antibodies against COX-2 (Abcam, Cambridge, MA, USA), inducible nitric oxygen synthase (iNOS) (Abcam), β-actin (Sigma), inhibitor of NF-κB (IκB)-α (Cell Signaling Technology, Beverly, MA, USA), or the phosphorylated form of IκB-α (Cell Signaling Technology) was performed using SDS-solubilized cellular proteins. Protein images were visualized by ImageQuant LAS-4000mini (Fujifilm, Tokyo, Japan) and quantified by Multi Gauge (Fujifilm).

### Measurement of lipid peroxide

RAW 264.7 cells were incubated with or without 10% hemolysate treatment in the medium mixed with an equal part of saline, 10% trehalose, or 10% maltose for 3 h. Collected cells were washed twice with PBS that was deoxidized by N_2_ aeration. The level of lipid peroxide (LPO) in the cells was measured using an LPO assay kit (Cayman) according to the manufacturer’s instructions.

### Angiographic and histological examinations in the rabbit single-hemorrhage model

The rabbit single-hemorrhage model was used as previously reported [24], with some modifications. Eighteen rabbits (weighing 2.8 kg to 3.4 kg) were anesthetized with inhalation of isoflurane. Fresh autologous arterial blood was drawn from the ear artery. After 1.0 mL of cerebrospinal fluid was removed, 1.0 mL of blood was immediately injected into the cisterna magna with the animal in a tilted, head-down, prone position. The animal was maintained in the same position for 60 min to allow the injected blood to coagulate around the basilar artery. Three hours after the blood injection, the animal received an injection of 1.0 mL of saline, 30% trehalose, or 30% maltose into the cisterna magna in the Saline, Trehalose, or Maltose groups, respectively. Prior to and 48 h after the induction of experimental SAH, a 4 F angiographic catheter was inserted into the left vertebral artery via the left femoral artery under fluoroscopy, and angiography of the basilar artery was performed by manual injection of 0.5 mL iohexol (350 mg of iodine/mL; Daiichi-Sankyo, Tokyo, Japan). After the angiography, mannitol (0.5 mg/kg/h; Yoshindo, Toyama, Japan) was administered intravenously to prevent increased intracranial pressure.

Angiographic images of the basilar artery were adjusted with TOSPAC (Total System Performance Assessment Code; Toshiba, Tokyo, Japan). The diameter of the basilar artery was measured as the mean diameters at three locations: distal, middle, and proximal. The length between the distal and proximal locations was set to 100 pixels on the computer monitor, and the middle location was set as 50 pixels from the distal and proximal locations. The real length per pixel was evaluated by measuring a 2 mm lead ball recorded at the same time as the basilar artery. These measurements were performed using a blinded method.

After perfusion fixation with 200 ml of PBS and 450 ml of 4% paraformaldehyde mixed with 50 ml of 25% glutaraldehyde, the basilar arteries were sampled. Paraffin sections (3 μm thick) of the basilar artery were prepared and then stained with hematoxylin and eosin (H&E). The cross-sectional area of the vessel lumen was determined by measuring the circumference of the vessel lumen and calculating the area as a generalized circle. These measurements were also performed using a blinded method.

### Rat femoral artery vasospasm model and immunostaining for cyclooxygenase-2, inducible nitric oxide synthase, and 7-ketocholesterol

We used the rat model for vasospasm established by Okada *et al.*[25], with modifications. After anesthesia with intraperitoneal injection of pentobarbital (50 mg/kg), both the left and right femoral arteries and veins in 60 rats (weighing 300 to 350 g) were covered with a vinyl chloride catheter (10 mm in length). Fresh autologous blood was drawn from the femoral vein and mixed 3:1 with saline or 15% trehalose solution; the final concentration of trehalose was 3.75%. The mixture was immediately injected into the strip of the left artery to fill it up. Saline alone was injected into the strip of the right artery as a control. At 1, 3, 5, 7, 10, and 20 days after treatment, cryosections (3 μm thick) of both femoral arteries were prepared and then stained with H&E. The cross-sectional area of the vessel lumen was determined by measuring the circumference of the vessel lumen and calculating the area as a generalized circle. The wall media thickness was averaged among four distant sites. These measurements were performed using a blinded method. For each parameter, the ratio of the value in the left artery (tested artery) to that in the right artery (control artery) was calculated.

The cryosections were incubated overnight at 4°C with or without primary antibodies against cyclooxygenase-2 (COX-2) (1:500; Abcam), inducible nitric oxide synthase (iNOS) (1:500; Abcam), and 7-ketocholesterol (7-KC) (1:500; NOF, Tsukuba, Japan). Antibody localization was detected with HRP-conjugated secondary antibody (Promega, Madison, WI, USA) and diaminobenzidine (DAKO, Carpinteria, CA, USA). The sections were counterstained with methylgreen.

### Statistical analyses

Data are expressed as means ± SD or median and range, and were analyzed for significance by Kruskal-Wallis test with Bonferroni’s correction or, when indicated, by Mann-Whitney’s *U* test. Values of *p* < 0.05 were considered statistically significant.

## Results

### Trehalose suppressed blood-induced inflammatory responses *in vitro*

To confirm the effect of hemolysate on the arachidonic acid cascade, murine macrophage-like cells (RAW 264.7) were treated with 10% concentrations of hemolysate for various time periods. This treatment induced a significant time-dependent increase in the production of the eicosanoid PGE_2_ (Additional file 1: Figure S1A). Similar effects were observed in the production of cysteinyl leukotrienes (data not shown). To determine whether trehalose attenuated the production of PGE_2_, trehalose was mixed with hemolysate (pre-treatment), with the culture medium, or with both. Compared with the saline control, the increment in PGE_2_ production induced by hemolysate was significantly reduced by pre-treatment of the hemolysate with trehalose or by the presence of trehalose in the culture medium (Additional file 1: Figure S1B). While pre-treatment was more effective than for culture medium treatment, the strongest effect was observed when the two were combined (Additional file 1: Figure S1B). By contrast, maltose, a structural isomer of trehalose with an α, α-1,4 glycosidic bond, had no effect on PGE_2_ production (Figure 1A). Similar results were obtained using HUVECs (Additional file 1: Figure S1C). Trehalose also suppressed the production of cysteinyl leukotrienes and prostaglandin D_2_, suggesting that trehalose treatment attenuates both COX and 5-lipoxygenase (5-LO) pathways, which metabolize arachidonic acid into prostaglandins and leukotrienes, respectively (Figure 1B and Additional file 1: Figure S1D). Indeed, western blotting analysis revealed that the production of COX-2 (an inducible isoform of COX) elicited by hemolysate was significantly diminished by trehalose treatment *in vitro* (Figure 1C).

Next we evaluated the effect of trehalose on the action of cytosolic phospholipase A_2_s (cPLA_2_s), which act upstream from COXs and 5-LO as rate-limiting enzymes in the arachidonic acid cascade, in hemolysate-treated cells. Treatment with hemolysate significantly increased the level of ^3^H]-arachidonic acid release when saline or maltose was used (Figure 1D). Trehalose treatment suppressed hemolysate-induced ^3^H]-arachidonic acid release (Figure 1D). To examine whether trehalose directly suppressed the enzyme activity of cPLA_2_, the phospholipid-cleaving activity of cPLA_2_s was evaluated in the presence of trehalose. The cPLA_2_s effectively cleaved the phospholipids in the presence or absence of trehalose (Additional file 1: Figure S1E), indicating that the reduction in lipid mediators by trehalose was not due to direct suppression of the enzyme activity of cPLA_2_s. The production of endothelin-1, which is a potent vasoconstrictor and which was reported to be increased by treatment with hemolysate in cultured endothelial cells [26], was also suppressed by trehalose in HUVECs (Figure 1E).

Induction of various pro-inflammatory cytokines has been observed in both hemolysate-treated cells and experimental SAH models [5,23,27]. To investigate the effect of trehalose on cytokine expression, the levels of TNF-α, IL-6, IL-1α, and IL-1β were measured in hemolysate-treated cultured cells. Trehalose significantly suppressed the hemolysate-induced productions of these cytokines (Figure 1F and G, and Additional file 1: Figure S1F and G). iNOS expression also plays an important role in the pathogenesis of cerebral injury after experimental SAH [20]. Thus, we examined the effect of trehalose on iNOS production induced by hemolysate treatment in cultured cells. Western blotting analysis showed that iNOS production was increased by hemolysate, but significantly reduced in trehalose-treated cells (Figure 1H).

Overall, these data suggest that trehalose inhibits the inflammatory responses induced by hemolysate *in vitro*.

### Trehalose suppressed activation of NF-κB induced by hemolysate *in vitro*

**Figure 2:**
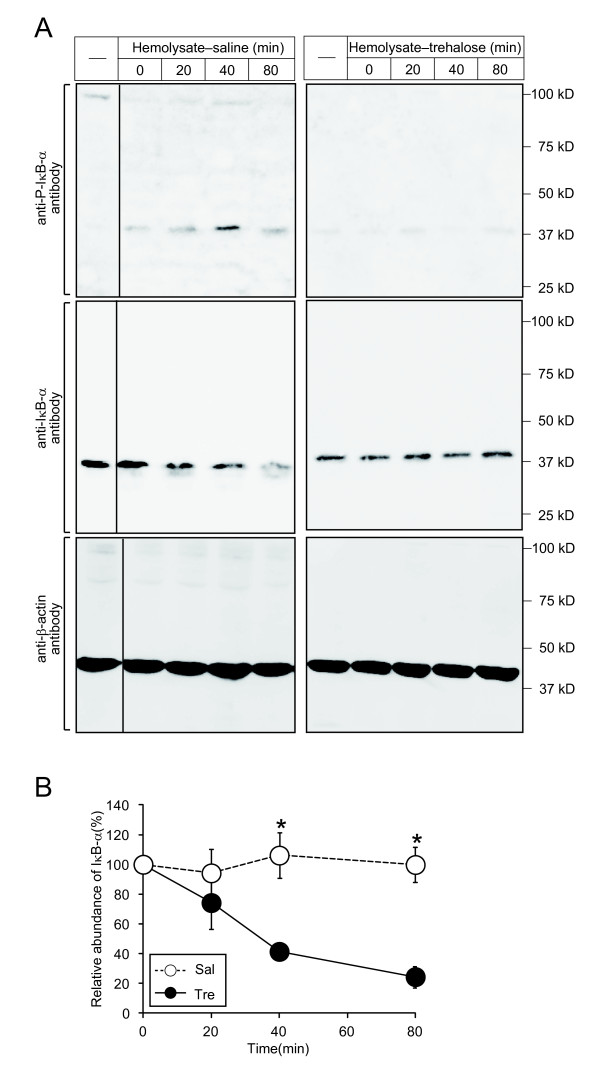
**Effects of trehalose on the NF-кB pathway activated by hemolysate treatment.** (**A**) Effect of trehalose on IкB-α phosphorylation and degradation in hemolysate-treated cells. RAW 264.7 cells were treated with 10% hemolysates or vehicle for 80 min. Cells were collected at the indicated time points, and then SDS samples were prepared. Amounts of phosphorylated IкB-α (P-IкB-α), total IкB-α (IкB-α), and β-actin were analyzed by western blotting. (**B**) The graph shows changes in the band intensities of total IкB-α, adjusted to the corresponding β-actin bands and calculated as intensity ratio relative to that of 0 min time-point, for 0, 20, 40, and 80 min. *P < 0.05 as compared to saline (Mann–Whitney‘s *U* test). n = 4 in each group. Whole experiments were repeated more than three times. All data are means ± SD.

NF-кB is activated in the arterial wall after experimental SAH, which may lead to induction of an inflammatory response including production of several cytokines [5]. We examined whether trehalose suppressed the activation of NF-кB in hemolysate-treated macrophage cells *in vitro*. NF-кB is activated by the phosphorylation and resultant degradation of IκB. Western blotting analysis showed that both the phosphorylation and degradation of IкB-α were induced by hemolysate treatment (Figure 2A and B). Both of these effects were clearly suppressed by trehalose treatment in the hemolysate-treated cells (Figure 2A and B). These data suggest that trehalose suppresses the inflammatory responses induced by hemolysate via inhibition of the canonical NF-кB pathways.

### Trehalose protected against hemolysate- and ROS-induced oxidative stress *in vitro*

**Figure 3:**
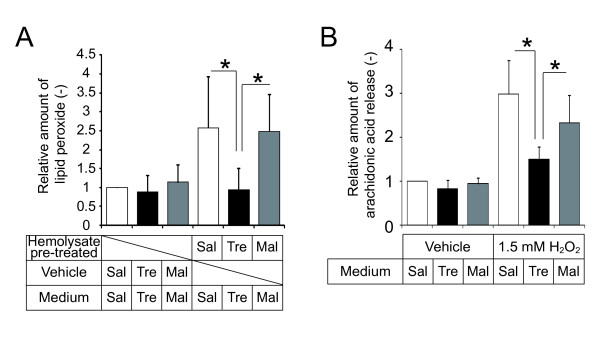
**Effects of trehalose on hemolysate-induced and ROS-induced oxidative stress in cultured cells.** As indicated in the graphs, the culture media, hemolysate, or both contained saline (Sal), 5% trehalose (Tre), or 5% maltose (Mal). (**A**) Measurement of lipid peroxide (LPO) using colorimetric analysis. RAW 264.7 cells were treated with or without 10% hemolysates for 3 h. LPO levels were measured by a colorimetric assay using ferrous iron molecules. n = 6 in each group. (**B**) Effect of trehalose on the release of arachidonic acid in H_2_O_2_-treated cells. RAW 264.7 cells were prelabeled with [^3^H]-arachidonic acid overnight and then treated with or without 1.5 mM H_2_O_2_ for 3 h. The radioactivity of the collected culture media was measured to quantify arachidonic acid release. (Kruskal-Wallis test). n = 4 in each group. All experiments were repeated more than three times. All data are shown as means ± SD. *P < 0.05 (Kruskal-Wallis test).

Oxidative stress, including lipid peroxidation, has also been shown to contribute to cell injury and progression of early brain injury and cerebral vasospasm after SAH [8,28]. Oxyhemoglobin released from the lysis of red blood cells is considered to play a major role in oxidative cell damage via lipid peroxidation in and around the cerebral artery [4,28]. Furthermore, superoxide anion radicals and hydroxyl radicals, which are generated by and released from oxyhemoglobin, are involved in lipid peroxidation [4]. Thus, we examined whether trehalose directly suppressed the generation of LPO. Colorimetric analysis confirmed that lipid peroxidation was stimulated by treatment with hemolysate in cultured cells (Figure 3A). Hemolysate-induced lipid peroxidation was significantly suppressed by trehalose, but not by maltose (Figure 3A). Next, to examine the effect of trehalose on the oxidative stress-induced arachidonic acid release, we performed an arachidonic acid release assay primed by H_2_O_2_ in cultured cells. H_2_O_2_ was previously reported to induce arachidonic acid release via the lipid peroxidation [29,30]. In our assay, trehalose significantly suppressed H_2_O_2_-primed arachidonic acid release, while there was no effect of maltose (Figure 3B). These data suggest that trehalose directly reduces oxidative stress, including lipid peroxidation, induced by hemolysate and ROS.

To elucidate the mechanism by which trehalose suppressed oxidative stress, we investigated the effect of trehalose on the scavenging of free radicals using electron spin resonance spectroscopy with the spin-trap reagent 5-(2,2-dimethyl-1,3-propoxy cyclophosphoryl)-5-methyl-1-pyrroline N-oxide (CYPMPO) [31]. There was no effect of trehalose on the generation of super oxide anions using a hypoxanthine/xanthine oxidase system, whereas trehalose reduced the generation of hydroxyl radicals using a Fenton reaction, suggesting that trehalose scavenged hydroxyl radicals (Additional file 1: Figure S2). However, the scavenging effect of hydroxyl radicals was also observed with maltose (Additional file 1: Figure S2). Thus, these data suggest that the suppression mechanism of blood- and ROS-induced lipid peroxidation by trehalose is not primarily the result of the scavenging of free radicals.

### Trehalose suppressed cerebral vasospasm after the onset of experimental SAH in the rabbit model

To test the effect of trehalose on cerebral vasospasm after experimental SAH, we used the rabbit single-hemorrhage model. In preliminary experiments, blood mixed with saline, 3.8% trehalose, or 7.5% trehalose was administered into the cisterna magna of rabbits (co-administration model). Angiography revealed that the blood-induced vasospasm was weaker in the blood + trehalose group than in the saline group (Additional file 1: Figure S3). Trehalose at a concentration of 3.8% was less effective for the suppression of vasospasm than that of 7.5% (data not shown).

**Figure 4:**
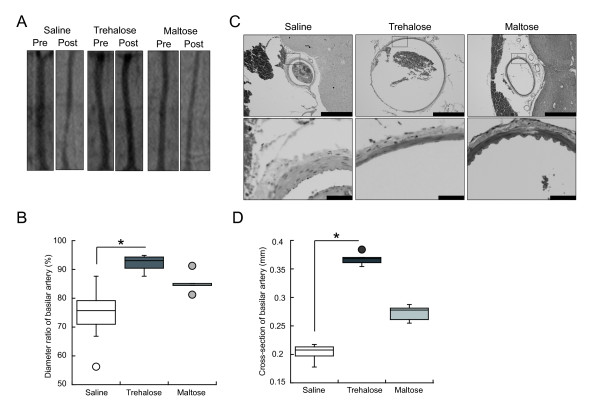
**Effects of trehalose on cerebral vasospasm in experimental SAH (post-administration model).** Saline, 30% trehalose, or 30% maltose was injected into the cisterna magna at 3 h after the experimental SAH induction. (**A**) Representative angiograms of the basilar arteries prior to (Pre) and 48 h after (Post) the induction of experimental SAH in rabbits. (**B**) The graph shows the diameter ratios of the basilar arteries after the induction of experimental SAH to the basilar arteries prior to the induction. *P < 0.05 (Kruskal-Wallis test). n = 6 in each group. (**C**) Upper panels, representative H&E-stained paraffin sections (3 μm thick) of basilar arteries. Scale bars, 1000 μm. Lower panels, higher magnifications of the squares in the upper panels. Scale bars, 100 μm. (D) The graph shows the cross-section of basilar artery in each group. *P < 0.05 (Kruskal-Wallis test). n = 6 in each group. All experiments were repeated more than three times. The boxes in graphs indicate the 25th and 75th quartiles and the central line is the median. The whiskers extend from the lowest to the highest value. Values outside the range of the whiskers are extreme values.

Next, to examine the effect of trehalose on cerebral vasospasm in a near-clinical setting, saline or trehalose at a final concentration of 7.5% was administered into the cisterna magna in a single dose at 3 h after blood injection (post-administration model). Blood in the cisterna magna was considered to be clotted at 3 h after blood injection. Similar to the case of co-administration of trehalose and blood, the vasospasm was significantly abrogated by post-administration of trehalose but not by post-administration of saline (Figure 4A and B). Maltose had a moderate inhibitory effect on vasospasm (Figure 4A and B). Histological analysis revealed that the characteristic features of vasospasm (reduction in the sectional area, thickening of the arterial wall, increase in endothelial cells, and corrugated internal elastic lamina) were observed in the basilar arteries of the saline- or maltose-injected animals, whereas the vasospasm was attenuated in the trehalose-injected animals (Figure 4C). Quantification of the cross-section of the basilar artery also revealed that post-injection of trehalose significantly suppressed the cerebral vasospasm (Figure 4D). Taken together, these data suggest that trehalose has a specific suppressive effect on the cerebral vasospasm caused by experimental SAH, even when administered after the onset of experimental SAH.

### Trehalose treatment abrogated blood-induced vasospasm, inflammatory responses, and lipid peroxidation in the presence of blood in the rat femoral artery model

**Figure 5:**
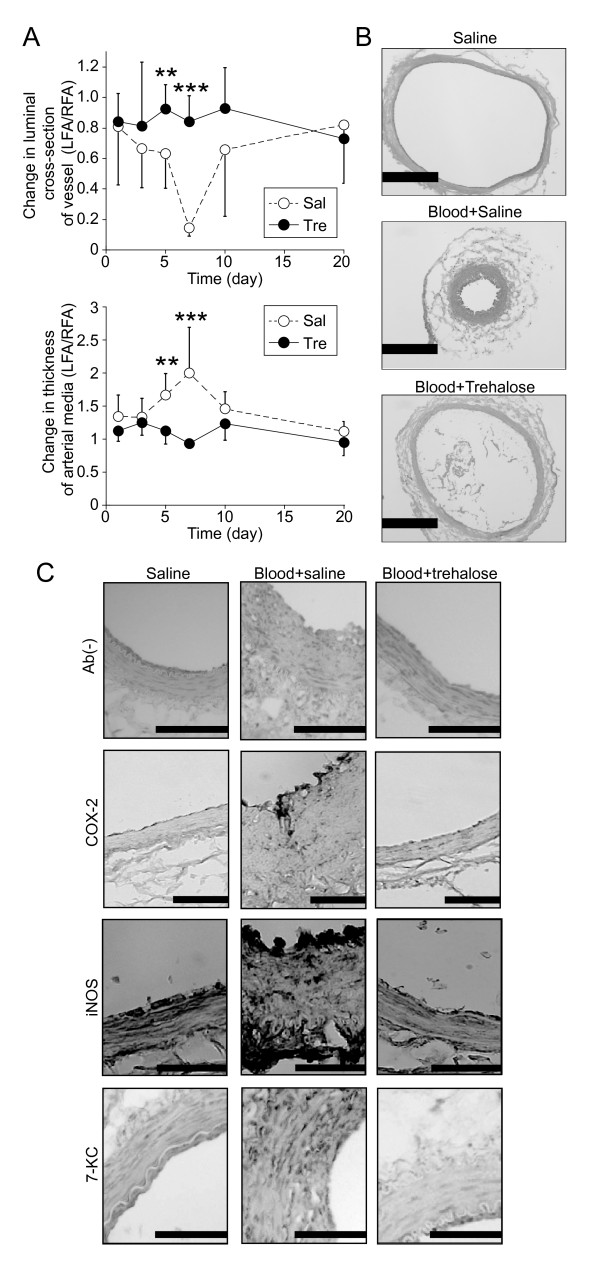
**Effects of trehalose on inflammatory responses, oxidative stress, and vasospasm in the presence of blood.** (**A**) Changes in the luminal cross-sectional area and thickness of the arterial media of femoral vessels exposed to saline or to blood containing either saline or trehalose for 1, 3, 5, 7, 10, and 20 days. The ratios of the luminal cross-sectional area and the thickness of the arterial media of femoral vessels exposed to blood containing either saline (Sal) or 3.75% trehalose (Tre) to those exposed to saline alone were calculated. **P < 0.01 and ***P < 0.005 * as compared with saline (Mann–Whitney‘s *U* test). n = 6 in each group. (**B**) Representative H&E-stained cryosections (3 μm thick) of femoral arteries exposed to saline alone (Saline) or to blood containing either saline (Blood + Saline) or 3.75% trehalose (Blood + Trehalose) at 7 days. Scale bars, 200 μm. (**C**) Immunohistological analysis in the rat artery vasospasm model. The cryosections (3 μm thick) were immunostained with or without antibodies (Ab) against COX-2, iNOS, or 7-ketocholesterol (KC) and then counterstained with methyl green. Scale bars, 20 μm. All experiments were repeated more than three times. All data are shown as means ± SD.

In our rabbit model studies, it was not clear whether trehalose directly suppressed vasospasm or whether it facilitated the recovery from vasospasm in arterial tissue exposed to blood. To address this question, we performed the time-course experiments using the rat femoral artery vasospasm model, which is analogous to clinical and experimental SAH in terms of the temporal evolution of vascular narrowing and morphological changes [25]. Control arteries exposed to saline for 7 days were similar in appearance to normal arteries (Figure 5B). Time-course experiments showed that the reduction in the sectional area and the thickening of the arterial walls both peaked at 7 days after treatment with a mixture of blood and saline (blood + saline), while these changes were suppressed by trehalose (final concentration of 3.75%) (Figure 5A). On day 7, the characteristics of vasospasm were clearly observed in the arteries treated with blood + saline (Figure 5B). By contrast, the arteries exposed to blood + trehalose exhibited minimal changes (Figure 5B). Trehalose was also effective at final concentrations of 1.875% and 7.5% (Additional file 1: Figure S4). These data suggest that trehalose directly prevented development of vasospasm induced by blood in the rat femoral artery model.

Next, we examined whether trehalose suppressed the activation of inflammatory responses and production of lipid peroxide induced by blood. We performed immunohistochemical analysis for the inflammatory markers COX-2 and iNOS in the rat models. In the blood + saline groups, endothelial cells in the intimal layer were stained with the COX-2 antibody, and endothelial cells in the intimal layer and fibroblasts in the adventitial layer were stained with the iNOS antibody (Figure 5C). By contrast, the immunostaining intensities for both COX-2 and iNOS were markedly diminished in the blood + trehalose groups and the negative control groups (Figure 5C). Immunohistochemical analysis also detected 7-KC, a marker for lipid peroxidation and a non-enzymatic oxidant of cholesterol [29], in smooth muscle cells of medial layer in the blood + saline group. By contrast, 7-KC production was not observed in the blood + trehalose group (Figure 5C). These data suggest that trehalose suppresses inflammatory responses and lipid peroxidation as well as the induction of vasospasm *in vivo*.

## Discussion

In the present study, we demonstrate that (1) trehalose suppressed hemolysate-induced inflammatory responses including activation of the arachidonic acid cascade and production of iNOS, pro-inflammatory cytokines, and endothelin-1 in cultured cells; (2) trehalose suppressed activation of the NF-κB pathway in the hemolysate-treated cultured cells; (3) trehalose protected cells from hemolysate- and ROS-induced lipid peroxidation in the cultured cells; (4) a single administration of trehalose after the onset of experimental SAH suppressed blood-induced vasospasm in the rabbit single-hemorrhage model; and (5) trehalose suppressed development of vasospasm, as well as inflammatory responses and lipid peroxidation in the presence of blood in the rat femoral artery model.

### Putative mechanisms of suppression of vasospasm by trehalose in subarachnoid space after the onset of experimental SAH

Our rabbit model studies showed that a single administration of trehalose even after the onset of experimental SAH suppressed cerebral vasospasm. In addition, we observed that trehalose suppressed development of vasospasm even in the presence of blood in the rat femoral artery model. These results suggest that trehalose, by remaining in the subarachnoid space, can suppress complications of SAH even if the blood clots are remained. It is unclear how trehalose exerts this anti-vasospasm effect on arterial tissue. Nevertheless, autoradioluminogram of the rabbits after intraperitoneal administration of radiolabeled-trehalose revealed that trehalose remained in intraperitoneal cavity and was relatively localized on the organ surface (likely through interaction with membrane) for several hours, while the trehalose was then transported to various other organs within 24 h (S. Kuribayashi, Personal communication). Therefore, similarly in the intraperitoneal space, trehalose may remain in the subarachnoid space for several hours and suppress cerebral vasospasm through its membrane-protective effect.

### Putative mechanisms of the anti-inflammatory effect of trehalose after treatment with blood

A number of reports have suggested an association between inflammatory responses and complications such as cerebral vasospasm after SAH [4,27,32]. We observed that hemolysate induced the production of several inflammatory mediators as well as the activation of NF-кB *in vitro*. We also confirmed that COX-2 and iNOS were produced in the rat femoral artery model, similar to the results with experimental SAH models previously described [19,20]. Our *in vivo* and *in vitro* studies clearly showed trehalose suppressed these inflammatory responses.

Production of the lipid mediators is mainly controlled by cPLA_2._ However, trehalose did not directly inhibit the enzyme activity of cPLA_2_, despite a decrease in production of lipid mediators and arachidonic acid release induced by hemolysate treatment in cultured cells. The Ca^2+^-independent PLA_2_ (iPLA_2_, group VI PLA_2_) is involved in arachidonic acid release induced by various stimulations and nicotine-induced contraction in the basilar artery via arachidonic acid metabolites [33,34]. Thus, iPLA_2_ may be involved in arachidonic acid release and vasospasm induced by blood, and trehalose may suppress the enzyme activity of iPLA_2_.

Production of pro-inflammatory mediators is partially regulated by the NF-кB pathway [35-37]. Our findings, in combination with those previously reported, suggest that trehalose suppresses the blood-induced inflammatory responses by inhibiting the NF-кB pathway. In support of these data, previous studies have demonstrated that trehalose prevented pro-inflammatory activation by endotoxic shock both *in vitro* and *in vivo*, potentially through several signaling pathways including the NF-кB pathway [13,14]. Thus, trehalose may be useful as a novel anti-inflammatory therapy.

We observed that pre-treatment of the hemolysate with trehalose was more effective than the presence of trehalose in culture medium for relieving blood-induced lipid mediator production *in vitro*. Sphingosylphosphorylcholine was recently identified as a pro-inflammatory factor after experimental SAH [38]. The anti-inflammatory effect of trehalose may be derived, at least in part, from inactivation of such pro-inflammatory factor (s). We also consider that the anti-inflammatory actions of trehalose are regulated by an identified or unidentified trehalose receptor expressed on the cell surface, and that this receptor attenuates activation of the signal transduction cascade such as the arachidonic acid cascade and the NF-кB pathway. Indeed, Taya *et al*. suggested that LPS-induced cytokine productions might be inhibited by trehalose via the T1R3 trehalose receptor in macrophage cells [39].

### Putative mechanisms of the antioxidant effect of trehalose after treatment with blood

The development of cerebral injury after SAH is triggered by oxidative stress including lipid peroxidation of the cell membrane [7]. Herein, we showed that trehalose suppressed the generation of lipid peroxidation induced by blood. Consistent with these data, trehalose was previously reported to suppress ROS-induced lipid peroxidation in yeast cells [40]. Trehalose may suppress lipid peroxidation by directly interacting with the membrane and thereby suppressing radical oxidation of unsaturated fatty acids [41]. Although trehalose appears to have an effect similar to free radical scavengers, the mechanism of action of trehalose clearly differs from that of free radical scavengers in that it protects the cell membrane.

### The effect of maltose on vasospasm, inflammation, and lipid peroxidation

While maltose had a moderate effect on vasospasm *in vivo* unlike trehalose, it was not effective in reducing blood-induced inflammation and lipid peroxidation. These results are in accordance with a previous report showing that maltose had no effect on an endotoxin-induced pro-inflammatory phenotype [14]. It is likely that the anti-inflammatory and antioxidant effects of trehalose are derived from its unique structural properties, not observed in maltose, that strongly interact with proteins and lipids through hydrogen bonding [42]. Further studies are required to determine the effect of trehalose on various pro-inflammatory factors and oxidants.

### Possible clinical use of trehalose after SAH

The most well established method for preventing the complications of SAH includes cisternal irrigation therapy with urokinase and intracisternal administration of tissue plasminogen activator. We found that injection of trehalose into the subarachnoid space after the onset of experimental SAH was able to inhibit vasospasm. Thus, in the clinical setting, we suggest that cisternal irrigation fluid is the optimal route of trehalose administration, and is anticipated to have an additional or synergistic therapeutic effect on complications of SAH when combined with existing therapeutic modalities.

### Study limitations

There are some limitations in this study. First, while we showed suppressive effects of trehalose on inflammation, oxidative stress, and vasospasm, we could not fully elucidate the relationship between these pathological mechanisms and the effects of trehalose. Secondly, although we consider that trehalose could be administered with cisternal irrigation therapy in the clinical setting, we examined the effect of trehalose on vasospasm *via* a bolus injection into the cisternal space, not cisternal irrigation. Thirdly, we only examined the inflammatory response and oxidative stress in the femoral arterial walls only. Finally, we did not evaluate any cerebral function and morphology such as mortality, neurological function, cerebral blood flow, and neuronal cell death in the rabbit model.

## Conclusions

We demonstrated that periadventitial treatment with trehalose can produce pleiotropic actions including inhibition of inflammatory responses, lipid peroxidation, and vasospasm. The presence of these multiple effects is advantageous compared with other drug candidates such as radical scavengers or anti-inflammatory reagents that exert only a single action. Trehalose has already been used as an organ preservation solution in clinical transplantation, and has no side effects. Thus, trehalose may be a potential therapeutic option for patients with SAH, either as monotherapy or in combination with other treatment modalities.

## Competing interests

The authors declare that they have no competing interests.

## Authors’ contributions

RE participated in the design of the study, performed *in vivo* experiments using rabbits and rats, and drafted the manuscript. NS participated in the design of the study, performed *in vitro* experiments, and drafted the manuscript. KK partly performed *in vivo* experiments using rabbits. FY performed immunohistological experiments. YK-K partly performed *in vivo* experiments using rats. TO performed *in vitro* PLA_2_ assay. AF, YK, MN, SS, MM, TS, UC, and NS participated in the study design and coordination and drafted the manuscript. All authors read and approved the final manuscript.

## Supplementary Material

Additional file 1 **Figure S1.** Effects of trehalose on other inflammatory markers in cultured cells. **Figure S2.** Scavenging effect of trehalose on superoxide anion and hydroxyl radicals *in vitro*. **Figure S3.** Effects of trehalose on cerebral vasospasm in experimental SAH (co-administration model). **Figure S4.** Effects of trehalose at various concentrations on blood-induced femoral artery vasospasm [43]. Click here for file
