# Supplementary material for: Trehalose treatment suppresses inflammation, oxidative stress, and vasospasm induced by experimental subarachnoid hemorrhage
Source: J Transl Med. 2012 Apr 30;10:80. doi: 10.1186/1479-5876-10-80 (PMC3422174; doi:10.1186/1479-5876-10-80)
Supplement: Additional file 1 — Figure S1. Effects of trehalose on other inflammatory markers in cultured cells. Figure S2. Scavenging effect of trehalose on superoxide anion and hydroxyl radicals in vitro. Figure S3. Effects of trehalose on cerebral vasospasm in experimental SAH (co-administration model). Figure S4. Effects of trehalose at various concentrations on blood-induced femoral artery vasospasm [43]. [file 1479-5876-10-80-S1.doc]

**Trehalose treatment suppresses inflammation, oxidative stress, and vasospasm induced by experimental subarachnoid hemorrhage.**

Ryosuke Echigo, Nobuyuki Shimohata, Kensuke Karatsu, Fumiko Yano, Yuko Kayasuga-Kariya, Ayano Fujisawa, Takayo Ohto, Yoshihiro Kita, Motonao Nakamura, Shigeki Suzuki, Manabu Mochizuki, Takao Shimizu, Ung-il Chung and Nobuo Sasaki

**Supplementary Figures**


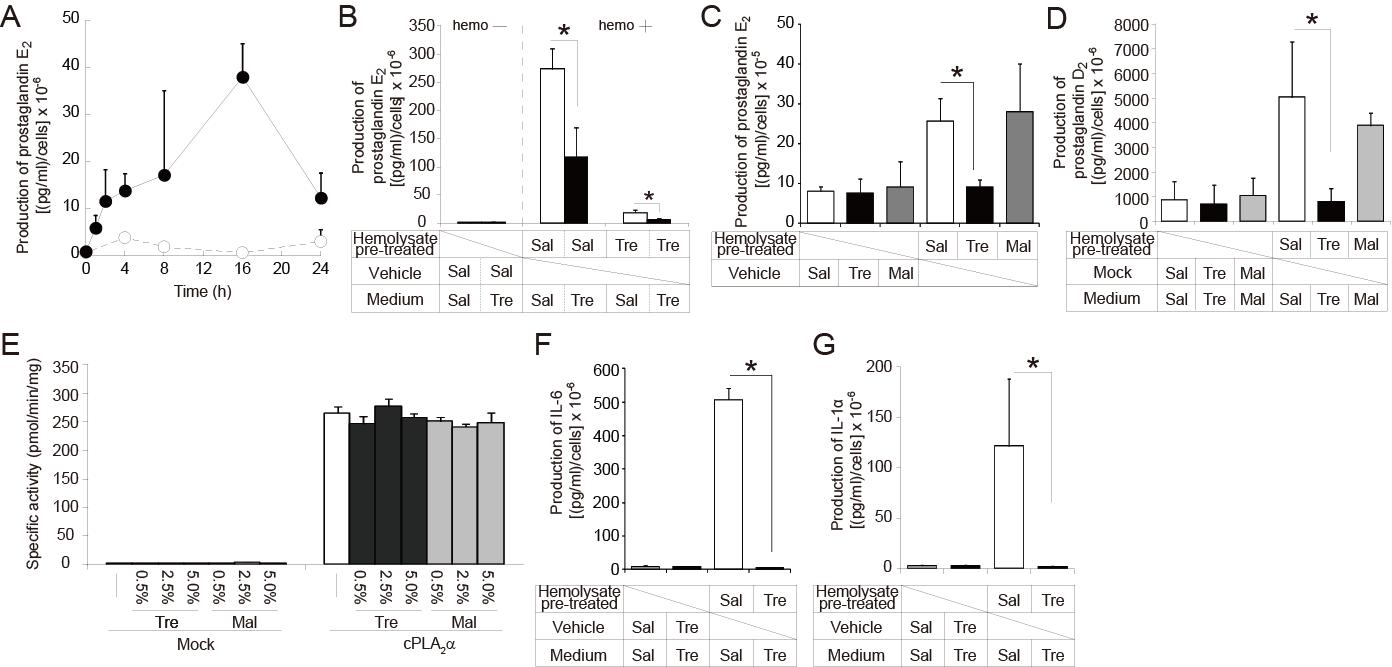


**Supplementary Figure 1 Effects of trehalose on other inflammatory markers in cultured cells**.

(**A**) Production of PGE_2_ in hemolysate-treated cells. RAW264.7 cells were treated with 10% hemolysate (black circles) or vehicle (saline, white circles) over the time course shown. PGE_2_ levels were measured by enzyme immunoassay (EIA). n = 3 in each group. (**B**) The anti-inflammatory effects of trehalose, when hemolysates were pre-treated with trehalose and when trehalose was mixed with culture media. RAW264.7 cells were treated with or without 10% hemolysates containing saline (Sal) or 7.5% trehalose (Tre), as indicated in the graph, in the presence of saline (Sal, black column) or 5% trehalose (Tre, white column) for 8 h. PGE_2_ levels were measured by EIA. n = 5 in each group. * *P* < 0.05 (Mann-Whitney‘s U test). (**C**) Effect of trehalose on induction of PGE_2_ in hemolysate-treated primary cells. HUVECs were treated with or without 10% hemolysates containing saline (Sal) or 7.5% trehalose (Tre), as indicated in the graph, for 18 h. PGE_2_ levels were measured by EIA. n = 4 in each group. **P* < 0.05 (Kruskal-Wallis test). (**D**) Effect of trehalose on induction of PGD_2_ in hemolysate-treated cells. RAW264.7 cells were treated with or without 10% hemolysates containing saline (Sal), 7.5% trehalose (Tre), or 7.5% maltose (Mal), as indicated in the graph, in the presence of saline (Sal), 5% trehalose (Tre), or 5% maltose (Mal) for 8 h. PGD_2_ levels were measured by EIA. n = 4 in each group. * *P*< 0.05 (Kruskal-Wallis test). (**E**) The cPLA_2_α activity determined as the amount of cleaved [^14^C]-labeled arachidonic acid. Centrifugal supernatants of HEK293 cell homogenates transiently transfected with an empty vector (mock) or a plasmid expressing cPLA_2_α were incubated with 1-palmitoyl-2-[^14^C]arachidonoyl-choline-containing micelles in the presence or absence of 0.5%, 2.5%, or 5.0% trehalose or maltose. Radioactivity in each sample was counted. (**F**) Effect of trehalose on induction of interleukin (IL)–6 in hemolysate–treated cells. RAW264.7 cells were treated with or without 10% hemolysates containing saline (Sal) or 7.5% trehalose (Tre), as indicated in the graph, in the presence of saline (Sal) or 5% trehalose (Tre) for 8 h. IL–6 levels were measured by EIA. n = 4 in each group. * *P*< 0.05 (Kruskal-Wallis test). (**G**) Effect of trehalose on induction of IL–1α in hemolysate-treated cells. RAW264.7 cells were treated with or without 10% hemolysates containing saline (Sal) or 7.5% trehalose (Tre), as indicated in the graph, in the presence of saline (Sal) or 5% trehalose (Tre) for 8 h. IL-1α levels were measured by EIA. n = 4 in each group. **P*< 0.05 (Kruskal-Wallis test). Data are representative of at least three independent experiments done in triplicate and shown as the mean ± standard deviation.


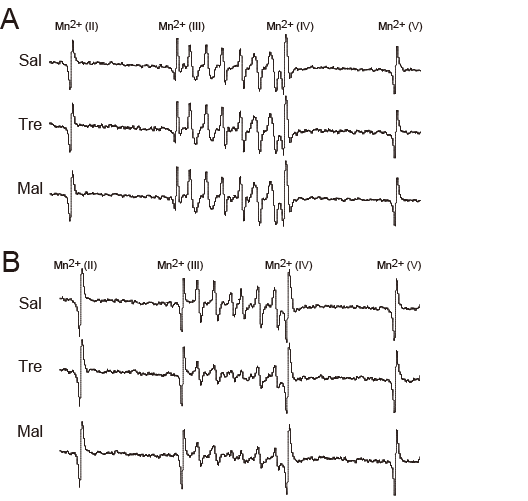


**Supplementary Figure 2 Scavenging effect of trehalose on superoxide anion and hydroxyl radicals *in vitro***.

Electron spin resonance (ESR) spectrum of the spin-trapped adduct of superoxide anion (**A**) generated by a hypoxanthine/xanthine oxidase system and hydroxyl radicals (**B**) generated by a Fenton reaction system. The ESR spectrum was measured in the presence of saline (Sal, upper), 5% trehalose (Tre, middle), or 5% maltose (Mal, bottom). 5-(2,2-Dimethyl-1,3-propoxy cyclophosphoryl)-5-methyl-1-pyrroline N-oxide (CYPMPO) was used as the spin-trapping reagent. All experiments were repeated more than three times and representative data were shown.


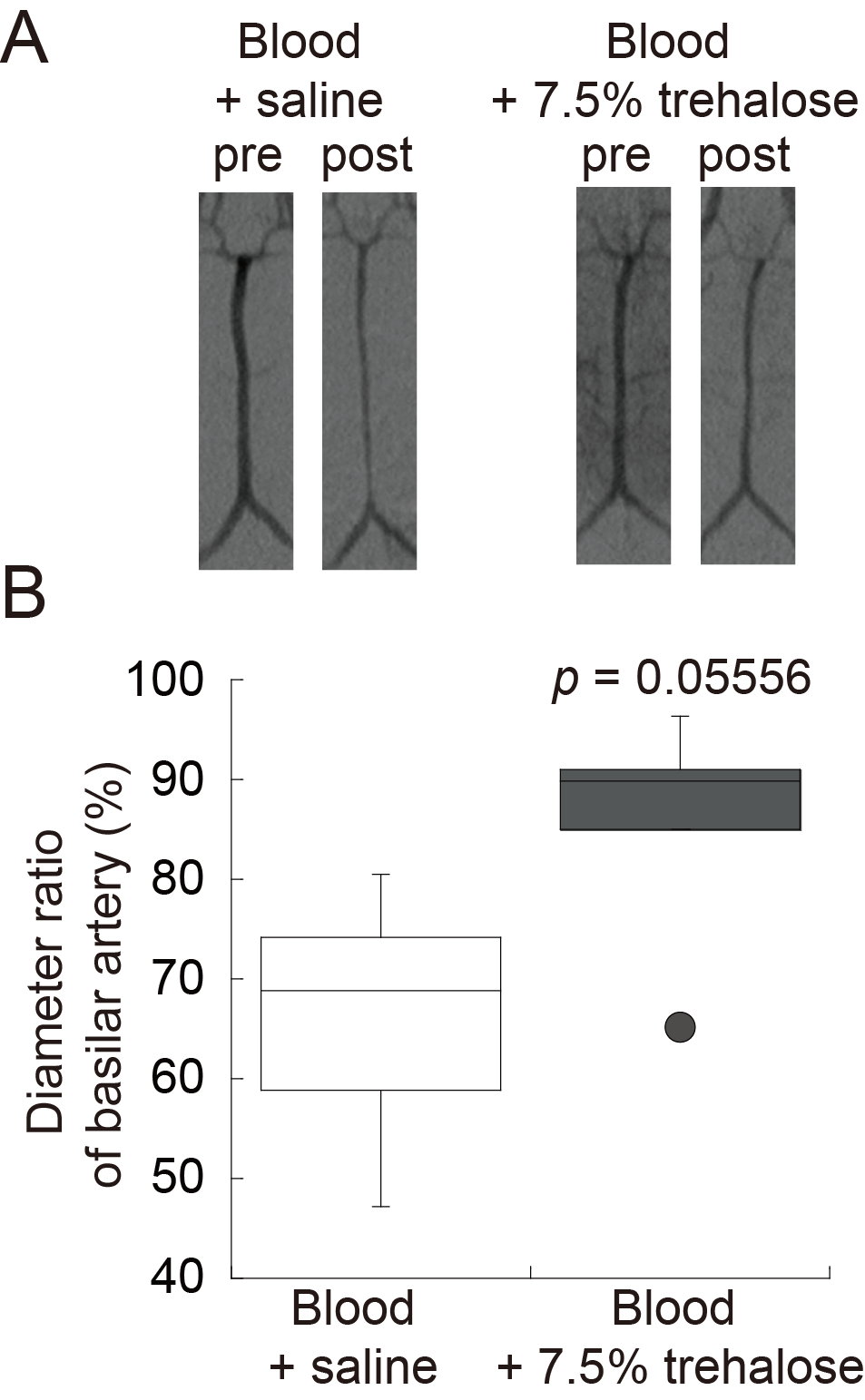


**Supplementary Figure 3 Effects of trehalose on cerebral vasospasm in experimental SAH (co-administration model)**.

(A) Representative angiograms of the basilar arteries prior to and 48 h after the induction of experimental SAH via the injection of arterial blood containing saline or 7.5% trehalose into the cistern magna. (B) The graph shows the diameter ratios of the basilar arteries after the induction of experimental SAH to the basilar arteries prior to the induction. **P* = 0.05556 (Mann-Whitney‘s U test). n = 6 in each group. All experiments were repeated more than three times. The box indicates the 25th and 75th quartiles and the central line is the median. The whiskers extend from the lowest to the highest value. Values outside the range of the whiskers are extreme values.


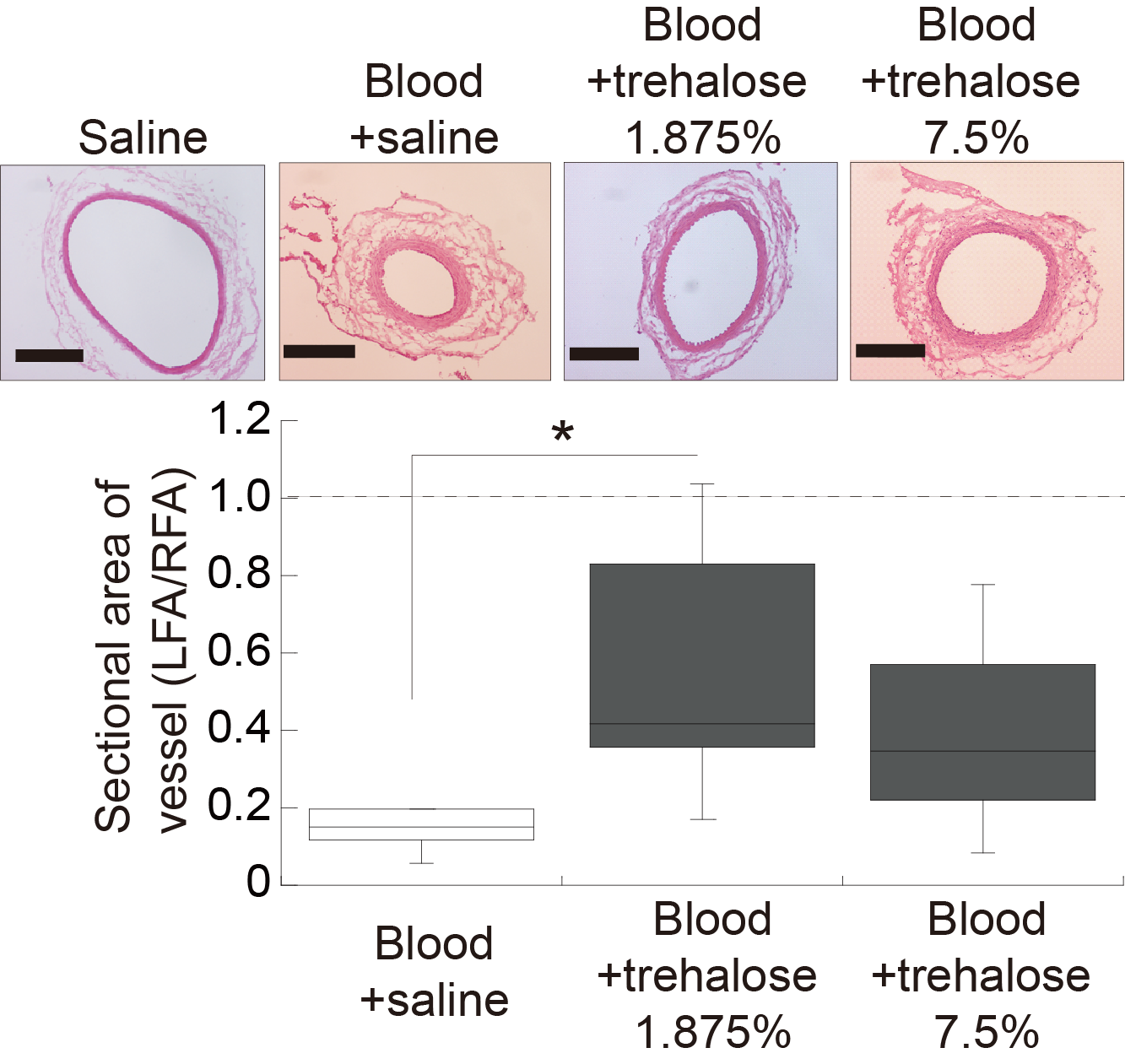


**Supplementary Figure 4** **Effects of trehalose at various concentrations on blood-induced femoral artery vasospasm**.

Histological and quantitative analysis of arterial vessels in the rat artery vasospasm model. The rat femoral arteries were exposed to saline or to blood containing either saline (blood + saline), 1.875% trehalose (blood + 1.875% trehalose), or 7.5% trehalose (blood + 7.5% trehalose) for 7 days. Upper panels, representative H&E–stained cryosections of femoral arteries. Scale bars, 200 μm. The sectional area of the arterial lumen was measured, and the ratio of the value in the left artery (blood + saline, white box; blood + trehalose 1.875% and 7.5%, dark gray box) to that in the right artery (saline) was calculated (lower panel). **P* < 0.05 (Kruskal-Wallis test). n = 6 in each group. All experiments were repeated more than three times. The box indicates the 25th and 75th quartiles and the central line is the median. The whiskers extend from the lowest to the highest value. Values outside the range of the whiskers are extreme values.

**Supplementary Methods**

**Transient expression and PLA_2_ assay**

The PLA_2_ assay was performed as previously described.^1^ HEK293 cells were transfected with pcDNA4HisMAX or pcDNA4HisMAX-cPLA_2_α using Lipofectamine Plus reagents (Invitrogen, Carlsbad, CA). Cells were harvested at 48 h after transfection, rinsed twice with PBS, frozen with liquid nitrogen, and stored at –80˚C until use. Thawed cells were suspended in homogenizing buffer [50 mM Tris-HCl (pH 7.5), 0.32 M sucrose, 4 mM DTT, 3 mM MgCl_2_, 5 mM EGTA, and 1x Complete™ protease inhibitor cocktail (Roche Diagnostics, Basel, Switzerland)] and homogenized with a sonicator (Ohtake Works, Tokyo, Japan). Centrifugations at 100,000 x *g* for 1 h were carried out for fractionation. PLA_2_ activity was measured using mixed micelles containing 1-palmitoyl-2-[^14^C] arachidonoyl-phosphatidyl choline (PerkinElmer Life Sciences, Boston, MA) and Triton X-100 in a molar ratio of 1:2 as a substrate; the final concentration of the substrate was 2 µM (54,000 dpm/reaction). The assay buffer contained 100 mM HEPES-NaOH (pH 7.5), 1 mg/ml BSA, 4.5 mM CaCl_2_, 1 mM DTT, and 0.5, 0.25%, or 5.0 % (w/v) trehalose or maltose. The reaction was started by the addition of enzyme sources, and the reaction mixtures were incubated at 37˚C for 30 min. The reaction was terminated by Dole’s reagent, and silica-gel powder was used to recover free fatty acid in the n-heptane layer. Radioactivity was counted with a liquid scintillation counter LS6500 (Beckman, Fullerton, CA).

**Measurement of electron spin resonance (ESR) by the spin-trapping method.**

A Fenton reaction system was used for the analysis of hydroxyl radical. This system consisted of phosphate–buffered saline, 0.1 mM ferrous sulfate, 1 mM hydrogen peroxide, 0.2 mM DETAPAC (dietylene triamine penta–acetic acid), and 1 mM 5-(2,2-dimethyl-1,3-propoxy cyclophosphoryl)-5-methyl-1-pyrroline N-oxide (CYPMPO) (Radical Research, Tokyo, Japan) with 5% trehalose, 5% maltose, and saline. The ESR spectrum was measured 1 min after addition of hydrogen peroxide. For the analysis of superoxide, a hypoxanthine (HX)/xanthine oxidase (XOD) system was used. This system consisted of 250 mM HX, 8 mU/ml of XOD, 1.1 mM DETAPAC, and 5 mM CYPMPO with 5% trehalose, 5% maltose, and saline. The ESR spectrum was measured 1 min after the addition of XOD. The ESR spectrum was recorded in a capillary tube at room temperature with a JEOL JES-FA spectroscope operating at X-band. Typical instrumental settings were as follows: incident-microwave 4 mW, modulation amplitude 0.2 mT, time-constant 0.03 s, and sweep time 1 mT/sec.

**Supplementary Reference**

43. Ohto, T., Uozumi, N., Hirabayashi, T. & Shimizu, T. Identification of novel cytosolic phospholipase A2s, murine cP_L_A2δ, ε, and ζ, which form a gene cluster with cP_L_A2β*. J Biol Ch*e**m 2**80, 24576-83 (2005).
